# *In Silico* Design and Experimental Validation of siRNAs Targeting Conserved Regions of Multiple Hepatitis C Virus Genotypes

**DOI:** 10.1371/journal.pone.0159211

**Published:** 2016-07-21

**Authors:** Mahmoud ElHefnawi, TaeKyu Kim, Mona A. Kamar, Saehong Min, Nafisa M. Hassan, Eman El-Ahwany, Heeyoung Kim, Suher Zada, Marwa Amer, Marc P. Windisch

**Affiliations:** 1 Informatics and Systems Department, Biomedical Informatics and Chemo-Informatics Group, Centre of Excellence for Advanced Sciences (CEAS), Division of Engineering Research, National Research Centre, Cairo, Egypt; 2 Centre for Informatics, Nile University, Shiekh Zayed City, Egypt; 3 Yousef-Jameel Science and Technology Research Centre, American University in Cairo, New Cairo, Egypt; 4 Biology Department, American University in Cairo, New Cairo, Egypt; 5 Immunology Department, Theodor Bilharz Research Institute, Giza, Egypt; 6 Faculty of Biotechnology, Misr University for Science and Technology, 6^th^ of October City, Egypt; 7 Hepatitis Research Laboratory, Institut Pasteur Korea, 696 Sampyung-dong, Bundang-gu, Seongnam-si, Gyeonggi-do, Republic of Korea; French-German Advanced Translational Drug Discovery Center, FRANCE

## Abstract

RNA interference (RNAi) is a post-transcriptional gene silencing mechanism that mediates the sequence-specific degradation of targeted RNA and thus provides a tremendous opportunity for development of oligonucleotide-based drugs. Here, we report on the design and validation of small interfering RNAs (siRNAs) targeting highly conserved regions of the hepatitis C virus (HCV) genome. To aim for therapeutic applications by optimizing the RNAi efficacy and reducing potential side effects, we considered different factors such as target RNA variations, thermodynamics and accessibility of the siRNA and target RNA, and off-target effects. This aim was achieved using an *in silico* design and selection protocol complemented by an automated MysiRNA-Designer pipeline. The protocol included the design and filtration of siRNAs targeting highly conserved and accessible regions within the HCV internal ribosome entry site, and adjacent core sequences of the viral genome with high-ranking efficacy scores. Off-target analysis excluded siRNAs with potential binding to human mRNAs. Under this strict selection process, two siRNAs (HCV353 and HCV258) were selected based on their predicted high specificity and potency. These siRNAs were tested for antiviral efficacy in HCV genotype 1 and 2 replicon cell lines. Both *in silico*-designed siRNAs efficiently inhibited HCV RNA replication, even at low concentrations and for short exposure times (24h); they also exceeded the antiviral potencies of reference siRNAs targeting HCV. Furthermore, HCV353 and HCV258 siRNAs also inhibited replication of patient-derived HCV genotype 4 isolates in infected Huh-7 cells. Prolonged treatment of HCV replicon cells with HCV353 did not result in the appearance of escape mutant viruses. Taken together, these results reveal the accuracy and strength of our integrated siRNA design and selection protocols. These protocols could be used to design highly potent and specific RNAi-based therapeutic oligonucleotide interventions.

## Introduction

### Hepatitis C Virus

**Fig 1:**
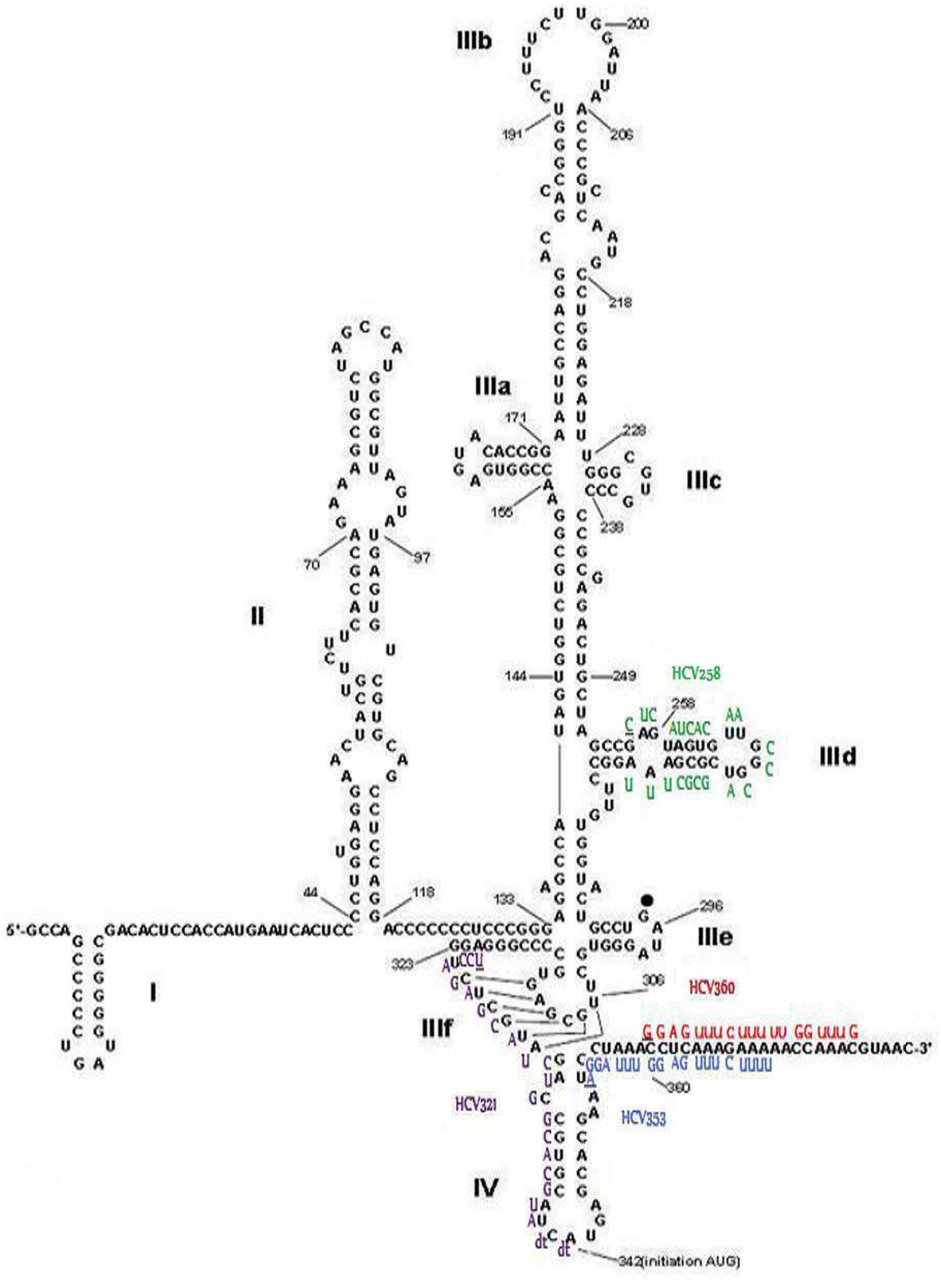
Scheme of HCV IRES and binding sites of siRNAs used in this study. The HCV IRES and the adjacent core sequence with the binding sites of the selected *in silico*-designed and reference siRNAs (HCV258, HCV 321, HCV353, and HCV360) used in this study are presented here. The first nucleotide of each individual siRNA binding to the IRES is underlined. Three of the most critical IRES loops (IIId, IIIf, and IV) have been targeted by HCV353 and HCV258 siRNAs. The 5′NTR scheme was modified with permission from the Nature Publishing Group (Lukavsky *et al*. [11]).

Approximately 200 million people worldwide are chronically infected with the hepatitis C virus (HCV). HCV is the major cause of acute hepatitis and chronic liver disease (e.g. cirrhosis and hepatocellular carcinoma). It is therefore also the leading indication for liver transplantation [1]. HCV is an enveloped, positive-stranded RNA virus belonging to the *Flaviviridae* family [2]. Seven major genotypes and numerous subtypes have been described; the genotype nucleotide sequences differ by as much as 30% [3,4]. The single-stranded 9.6-kb genome consists of a single open reading frame flanked at the 5′ and 3′ ends by highly structured and conserved non-translated regions (NTRs). These NTRs are important for viral translation and viral replication [5]. The approximately 340-nucleotide NTR sequence at the 5′ end contains an internal ribosome entry site (IRES) that directs translation independent of a cap-structure. The viral polyprotein is co- and post-translationally processed into 10 viral proteins (core, E1, E2, p7, NS2, NS3, NS4A, NS4B, NS5A, and NS5B). The highly conserved HCV 5′NTR and its IRES (Fig 1) are characterized by the formation of complexes with the host-cell small ribosomal subunit (40S) and eukaryotic initiation factor (eIF). These complexes result in the recognition of the viral RNA start codon, and the initiation of viral protein synthesis [6]. Studies of the structure and mechanisms by which the IRES functions have highlighted its potential as a suitable target for drug discovery [7] because this sequence is highly conserved throughout most HCV strains and has a vital role in the viral life cycle [8]. The 5′NTR is composed of four secondary structured domains (I, II, III, and IV) that were predicted based on thermodynamic calculations, mutational analysis, and phylogenetic considerations [9]. The structure was further studied using electron microscopy (EM) and nuclear magnetic resonance spectroscopy techniques. The results revealed a more complex tertiary structure formation between the IRES, which was used for further subdomain classification [10]. The IRES-dependent mechanism for translation initiation is shared by other viruses and by some eukaryotic RNAs. However, the structure of the HCV IRES is likely to be different from that of human mRNA IRES structures. Therefore, this RNA motif and its complexes with the 40S ribosomal subunit and eIF3 may be attractive targets for new antiviral agents.

It is now possible to examine all of the steps in the viral life cycle. Entry, viral RNA replication, infectious viral particle formation (packaging, assembly, and release), and *in vivo* infection can be investigated using pseudoparticles (HCVpp) [12], subgenomic replicon cells [13], infectious HCV cell culture system (HCVcc) [14], and transgenic mice [15], respectively. Among these approaches, the development of the subgenomic replicon system represents an important advance because it facilitates evaluation of potential antivirals using a cell culture system. The HCV subgenomic replicon consists of an HCV RNA. The HCV structural protein region is replaced by the neomycin phosphotransferase gene, and translation of the viral non-structural proteins (NS3 to NS5) is directed by the encephalomyocarditis virus (EMCV) IRES element flanked by the 5′ and 3′NTRs. Stable HCV RNA replication has been established in various cell lines (i.e., liver and non-liver, and human and non-human). These cell lines are excellent tools for study of the HCV life cycle and validation of novel antivirals [13,16,17,18].

Despite increasing efforts to develop novel drugs that are effective against HCV, patients are mainly treated using a virus-nonspecific combination therapy of pegylated interferon alpha (PEG-IFNα) and ribavirin. This treatment is associated with severe side effects and is effective in only 50–60% of patients infected with the HCV genotype 1 [19]. Development of direct-acting antivirals (DAAs) that target the viral NS3/4A protease resulted in an expectation that use of IFNα therapy could be stopped. Unfortunately, therapy with these first-generation protease inhibitors is frequently accompanied by severe adverse effects, has a low resistance barrier, and the administration regime is inconvenient to patients [20]. More recently, the US Food and Drug Administration (FDA)-approved drugs targeting HCV NS5A and NS5B, and second-generation protease inhibitors, are being used in the clinical setting. This change has resulted in improved antiviral efficacies and fewer reported side effects. However, the high economic burden limits access to therapy and, eventually, as for all drugs targeting RNA viruses, drug resistance will likely become an issue. These unmet medical needs encouraged us to identify new viral drug targets with a higher genetic barrier to resistance and effectiveness against all HCV genotypes. Consequently, we considered the use of small interfering RNAs (siRNAs) that target highly conserved regions of the HCV genome. We anticipate that HCV-specificity could further increase sustained virologic response rates (SVRs), reduce the appearance of viral escape mutations, and lower the economic burden of therapy.

### RNA Interference

RNA interference (RNAi) is involved in many biological processes. A series of *in vitro* and *in vivo* studies have revealed the importance of the regulatory roles of RNAi for control of gene expression [21]. Briefly, the mechanism of RNAi is as follows: siRNAs are nucleotide duplexes 21 to 23 base pairs in length that are incorporated into a protein complex (i.e., the RNA-induced silencing complex [RISC]). RISC forms an effector complex with the target RNA in a sequence-specific manner, which leads to its degradation and results in the silencing of mRNA expression [22]. Human pathogens are ideal targets for RNAi because they carry unique genome sequences, thereby minimizing unintentional off-target effects. Therapeutic effects of RNAi *in vivo* towards human pathogenic viruses have been reported previously. For example, in hepatitis B virus (HBV) and human papillomavirus, treatment with HBV-specific and E6-specific siRNAs leads to HBV DNA reduction [23] and inhibition of tumor growth, respectively [23]. Likewise, several studies have shown long-term inhibition of influenza virus replication. *In silico* (computational) studies for optimal design, followed by selection with experimental validation, have been performed to further improve the siRNA design [24]. The HCV 5′NTR has been a central focus of antiviral research because it is highly conserved among different isolates and contains an IRES element essential for the cap-independent translation of the viral RNA [25]; a single inhibitor may be effective against multiple, if not all, genotypes. In addition, due to the lack of a proofreading function in the viral polymerase (NS5B), statistically, every HCV RNA genome incorporates one mutation per genome per replication cycle, which leads to a complex mixture of genetically distinct, but closely related, variants referred to as a “quasispecies”. With regard to the quasispecies populations that emerge after infection within a patient, choosing a highly conserved target site reduces the probability of escape mutations and prevents the development of viral resistance to therapy.

### siRNA Design, Scoring, and Algorithms

The use of an integrative protocol for designing potent siRNAs is of extreme importance. *In silico* siRNA design facilitates the prediction of highly potent siRNAs before the synthesis and the subsequent biological assay of the selected siRNAs. Many issues must be considered when siRNAs are designed, including sequence space conservation, the siRNA sequence, and siRNA and target mRNA structure constraints. In addition to the specificity and efficiency of the silencing potency of siRNAs, it is important to consider the biological aspects, such as intracellular siRNA stability and avoidance of immunostimulation by siRNAs [26,27].

Since 2004, the basic guidelines for design of functional siRNAs have been outlined in previous reports [28,29,30,31,32]. Various algorithms have been designed to apply several of these primary guidelines to the design of siRNAs for a given sequence target. Advances in siRNA design and scoring include the use of machine-learning algorithms (artificial neural networks, support vector machines, linear regression models, genetic programming, and Bayesian inference) [33,34]. Most of these techniques include position-specific sequence features; some also include thermodynamics and target accessibility considerations [35,36,37,38].

The recently developed”MysiRNA-Designer” is an automated pipeline for siRNA design and selection workflow [39]. This method considers target RNA variations, siRNA and RNA target accessibility, off-target effects, siRNA antisense differential-end thermodynamic instability, siRNA folding, and target accessibility. In this article, our integrative siRNA design includes various bioinformatics steps, such as multi-score based filtration, two-step off-target filtration, and different target accessibility evaluation. We combined this bioinformatics workflow with the application of the MysiRNA-Designer and designed siRNAs that specifically target highly conserved regions in the IRES element within the 5′NTR of the HCV genome.

Here, we report on the use of an integrated approach combining the MysiRNA-Designer with various bioinformatics steps to design highly potent and selective siRNAs that target the HCV IRES element and adjacent core sequences. Multiple parameters, such as multiple transcript filtration, target accessibility, and off-target filtration analysis, were integrated. Selected siRNAs were validated using time- and dose-dependence experiments and were compared with previously described siRNAs that targeted the HCV IRES. Replicon cells of hepatoma and hepatoblastoma origin, replicating HCV genotype 1 or 2 subgenomic RNAs, and Huh-7 cells infected with patient-derived HCV genotype 4 isolates, were used to precisely quantify the inhibition of viral RNA replication following siRNA transfection. Our results indicated that one of our *in silico*-designed siRNAs exhibited very potent antiviral efficacy against HCV genotypes 1, 2, and 4, even at time points soon after siRNA transfection (24 h) and at low concentrations (0.1 nM). There was also no apparent cytotoxicity, which suggested that the designed siRNAs were HCV-specific. Ultimately, this work represents an integrative methodology that can be used to design siRNAs *in silico*, and also validated these siRNAs by demonstrating efficient HCV RNA replication inhibition in various cell lines with different HCV genotypes.

## Materials and Methods

### *In Silico* siRNA Design

**Fig 2:**
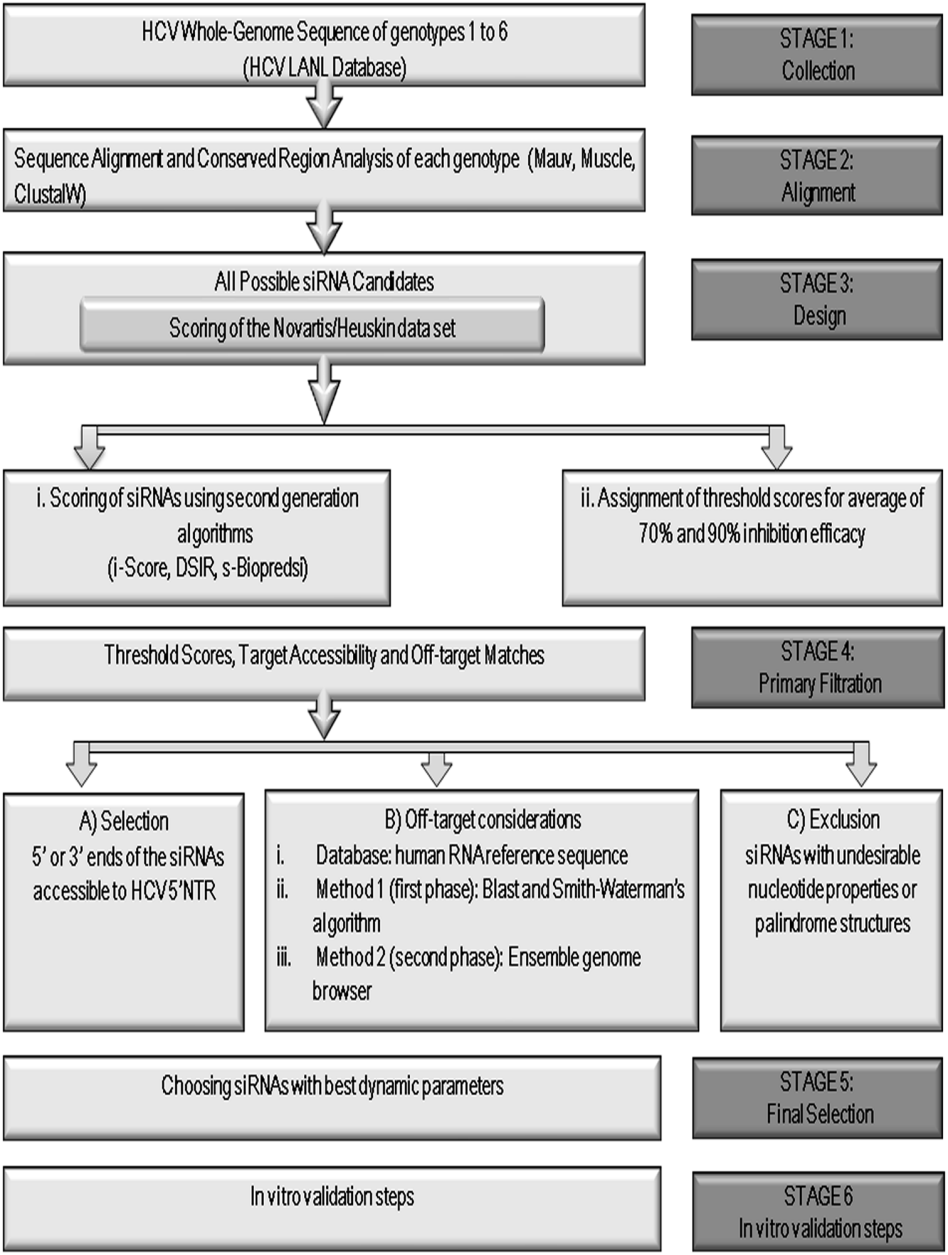
Flowchart of steps and methodology for *in silico* siRNA design. Steps 1–3: HCV IRES sequences of different genotypes were selected and aligned. All possible siRNAs were designed and sorted according to the parameters, as depicted. The candidates were scored according to first- and second-generation algorithms, and the 70% and 90% experimental threshold inhibition scores were determined. Step 4: Multi-stage filtering of siRNAs based on threshold scores, off-targets, palindromes, and repeat-motifs was performed, followed by selection of siRNAs with terminal ends mapped to a crucial IRES loop. Step 5: Additional off-target seed-region matches were filtered, and siRNAs with optimal thermodynamic properties were selected.

The methodology used for design of the siRNAs is presented in the following paragraphs (1) to (5) and outlined in Fig 2. The characteristics of the siRNAs used in this study and the thermodynamic properties of our *in silico*-designed siRNAs are presented in Table 1 and S1 Table, respectively.

**Table 1:** Features of siRNAs validated in HCV replicon assays. siRNA sequences targeting HCV IRES subdomains III and IV.

HCV siRNA^1^	siRNA sequence (sense)^2^	Target IRES subdomain	Position	Degree of conservation^3^	Thermodynamic properties^4^	Scoring^5^
HCV258	5'GUAGUGUUGGGUCGCGAAAdTdT3'	IIId	258–266	89.5%	-38.7	>90% threshold
HCV321	5'AGGUCUCGUAGACCGUGCAdTdT3’	IIIf and IV	321–338	100%	-43.2	< 90% threshold
HCV353	5'UCCUAAACCUCAAAGAAAAdTdT3'	IV	353–371	100%	-32.1	>90% threshold
HCV360	5'CCUCAAAGAAAAACCAAACdTdT3’	IV	360–378	100%	-31.4	< 90% threshold

^1^ siRNA numbering corresponds to the first siRNA nucleotide binding to the sequence of HCV genotype 1b Con1 strain.

^2^ siRNA sequence with dT (deoxythymidine overhang).

^3^ Degree of conservation (%) of each siRNA-targeted sequence between six HCV genotypes (Kroenke *et al*.)

^4^ Whole ΔG

^5^Comparison to the three second generation scoring tools (i-SCORE, s-Biopredsi and DSIR)

### Sequence Download, Alignment, and Analysis

All complete HCV whole-genome reference sequences were downloaded from the HCV LANL database (http://hcv.lanl.gov/content/index) for each of the six genotypes (1–6), and for all of the subtypes available in the database [40]. To identify highly conserved regions in the viral RNAs, whole-genome alignments for each genotype were performed separately using Muscle, and ClustalW2.0, software [41]. The BioEdit sequence alignment editor was used to construct consensus sequences for each HCV genotype [42].

### siRNA Design, Scoring, and Selection Steps

All possible siRNAs were designed and scored using i-SCORE Designer, siVirus, and CAPSID software [34,43,44]. i-SCORE calculates nine different siRNA-designing algorithm scores (i.e., Ui-Tei [30]‚ Amarzguioui [31]‚ Hsieh [32]‚ Takasaki [29]‚ s-Biopredsi [45]‚ i-SCORE‚ Reynolds [28]‚ Katoh [46] ThermoComposition19 & ThermoComposition21 [47] ‚ and DSIR [48]). i-SCORE‚ s-Biopredsi‚ and DSIR have higher correlation coefficients than the primary algorithms. After the design stage, we implemented a consensus multi-score threshold filtration layer by defining threshold accepted scores for these tools. We triaged the designed siRNAs through all of these thresholds and selected only those siRNAs that exceeded all of them. We, thus, integrated the results from the different scoring tools to select siRNAs with the desired characteristics.

### Thermodynamics and Target Accessibility

EmBOSS explorer was used to filter out palindromic sequences in the siRNAs of a length longer than four nucleotides [49]. We selected siRNAs with high threshold scores and ones that mapped to 90% inhibition of viral transcripts. We then performed expression rate analysis for their target accessibility using the RNAxs tool [50]. siRNAs targeting accessible regions of the IRES (loop structures) either at the 5’ or 3’ ends of the siRNA were then selected. siRNAs that mainly targeted the IRES stem base pairs were filtered out because they had a reduced probability of binding. Other important thermodynamic features (e.g., whole stacking energy, terminal dinucleotide thermodynamics, siRNA free energy, maximum length of GC stretches, and GC%) were analyzed using i-SCORE [34]. The RNAxs tool was used to predict target accessibility.

### Off-Targets

A two-phase search for off-targets was performed. In the first phase, near-complete matches to the Human NCBI Reference mRNA sequence database [51] were filtered out using the two database similarity-searching algorithms BLAST [52] and Smith-Waterman, as implemented in ParAlign [53,54]. Both algorithms were used to increase the validity percentage of our off-target search. The second phase of the off-target search was performed to filter out siRNAs with seed-region complementarity to human mRNAs [55]. This siRNA exclusion was based on recent observations that exact complementarity at the 3′NTR region with nucleotides 2 to 7 at the 5’ end of the antisense strand of the siRNA (seed region) increases the probability of binding in an miRNA-like manner [56,57]. The Ensemble genome browser [58] was used to reject siRNAs with matches within the designed siRNAs seed region at 3′NTR of the various human gene transcripts.

### Final Optimization and Selection of siRNAs

The selection of siRNAs was optimized by choosing siRNAs with the best overall parameters for siRNA ΔG and differential-end instability as a significant thermodynamic feature. The siRNAs with the highest predicted efficacy, specificity, and ability to target the accessible loops of the HCV IRES region and adjacent sequences were selected (S1 Table).

### Cell Culture

Naïve and stably HCV-replicating HuH6 [59] and Huh-7 cells [60] were maintained in Dulbecco’s modified Eagle’s medium (WelGENE), supplemented with 10% heat-inactivated fetal bovine serum (PAA), 2 mM L-glutamine, nonessential amino acids, 100 U penicillin, and 100 μg streptomycin at 37°C and 5% CO_2_. These supplements and G418 were used for the selection of replicon cell lines and were purchased from Gibco Life Technologies. Huh-7 replicating HCV genotype 1b (Con1) replicons (pFKi389neoEI/NS3-3’_dg_Con1 NS5Aaa383_emGFP), and Huh-7 luciferase replicon cells (pFK-luc-ubi-neo-SG-Con1) and replicating HCV genotype 2a (JFH-1) (pFK-luc-ubi-neo_SG-JFH-1) replicons were kindly provided by Dr. Ralf Bartenschlager, University of Heidelberg, Germany.

### Plasmid Construction

Plasmid pFKi389neoEI_NS3-3’_dg_JFH-1 NS5Aaa383_emGFP (GFP replicon) was constructed by replacing the red fluorescent protein (RFP) gene in pFKi389neoEI_NS3-3’_dg_JFH-1_NS5Aaa383_RFP [61] with the gene encoding emGFP, using PmeI and XbaI restriction sites flanking the RFP gene.

### Generation of Cell Lines Replicating HCV Subgenomes

*In vitro* RNA transcription and RNA transfection were performed as previously described [13,62]. Briefly, to generate cell lines with HCV subgenomes, 1x10^6^ HuH6 or Huh-7 cells were electroporated with 5 μg *in vitro* transcripts of pFKi389neoEI/NS3-3’_dg_JFH-1_NS5Aaa383_emGFP. The electroporation conditions were 975 μF and 270 V using a Gene Pulser system (Bio-Rad) and a cuvette with a gap width of 0.4 cm (Bio-Rad). Cells were seeded into three, 10-cm diameter dishes. After 24 h of incubation, cells were selected at various G418 concentrations. Approximately 4 weeks after electroporation, colonies were pooled and expanded for further analysis. Selected cell lines replicating HCV subgenomes (replicon cells) were maintained at 1 mg/mL G418.

### RNA Interference and Determination of HCV RNA Replication

The siRNAs that were used in this study to target the HCV 5’NTR (Table 1) were custom-made (Dharmacon, ThermoScientific). A non-targeting siRNA control (Scramble) #1: D-001210-01-20 (Dharmacon, ThermoScientific) was also used. HuH6 (JFH-1) or Huh-7 (JFH-1 or Con1) GFP replicon cells were plated in complete DMEM without G418 at 2,500 or 3,000 cells per well (384-well format; Greiner), respectively. At 20 h post cell plating, siRNAs were introduced into the cells at defined concentrations (0.1, 0.25, 0.5, 1, 5, 10, 25, or 50 nM) by forward transfection using Lipofectamine2000 (Invitrogen) according to the manufacturer’s instructions. At defined time intervals post siRNA transfection (24, 48, and 72 h), the cell culture medium was removed and the cells were fixed using 3.7% PFA (USB Corp.). The cells were then washed with phosphate buffered saline (PBS), and cell nuclei were stained using 10 μg/mL Hoechst 33432 (Sigma)/PBS. Images were acquired using an automated ImageXpressUltra microscope (Molecular Devices Inc.) at 20x magnification and at two wavelengths. The wavelength 488 nm was used to detect GFP (marker for HCV RNA replication) and the wavelength 405 nm was used to detect Hoechst-stained cell nuclei (to determine total cell number). The images were analyzed using MetaXpress Cell Scoring software (Molecular Devices) to determine the percentage of HCV-positive cells (GFP-positive cells x 100/total cell number). For Huh-7 (JFH-1 or Con1) replicons expressing *Firefly* luciferase, cells were plated at 3,000 cells per well (384-well format; SPL) and transfected with siRNAs using Lipofectamine RNAiMax (Invitrogen) under the same conditions as described for GFP replicon cells. At a defined time interval post siRNA transfection, *Firefly* luciferase activity (marker for HCV RNA replication) and cell viability were assessed using Bright-Glo reagent (Promega) and CellTiter-Glo reagent (Promega), respectively. Bioluminescence was quantified using a Victor3 Multilabel plate reader (PerkinElmer). All siRNA knockdown experiments were repeated at least twice and were analyzed using Excel (Microsoft) and Prism (GraphPad Software) software. The mean values for the percentage of GFP positive cells and for relative light units (RLUs) were normalized to the mean value of the scramble siRNA control.

### Curing of HCV Replicon Cells with siRNAs

Huh-7 cells replicating subgenomic HCV JFH-1 replicons and expressing an NS5A-GFP fusion protein, were seeded at 4x10^5^ cells/well into 6-well plates. The cells were treated twice per week for 2 months with 50 nM siRNA, either individually (scramble, HCV321, or HCV353) or in combination (HCV321 and HCV353), without G418 selection. siRNA transfection was then discontinued, replicon cells were re-seeded into 10-cm diameter dishes, treated with 500 μg/mL G418 for 4 weeks and resistant cell clones were stained with crystal violet (100 mL solution of 0.5 g crystal violet [0.5% w/v], 25 mL methanol, and 75 mL D.W.).

### Infection Experiments with HCV Genotype 4 Patient Isolates

#### Serum sample selection

HCV sera samples were collected from chronically infected patients (Theodor Bilharz Research Institute Hospital, Egypt). The HCV diagnosis was based on detection of anti-HCV antibodies in sera using a recombinant HCV antigen-based test (Axiom Diagnostic). HCV-antigen-positive sera were also confirmed *via* RT-PCR, followed by nested PCR. Briefly, total RNA was isolated from the sera of four patients using the RNeasy MiniKit (Qiagen). cDNA was generated using the OneStep RT-PCR kit (Qiagen) according to the manufacturer’s recommendations. Forward primer specific to 5'NTR (5’ AAC TAC TGT CTT CAC GCA GAA 3’) and reverse primer specific to the C-hydrophobic region of E1 (5’ TGC TCA TGG TGC ACG GTC TA 3’) were used in the first amplification.

Ethics statement: For this study patient derived HCV was used. The ethics committee of the Theodor Bilharz Research Institute (TBRI) in Egypt and adhered to the tenets of the Declaration of Helsinki approved this consent procedure to use patient derived material. Written consent, stored at the TBRI, was obtained from participants for the use of their blood in this study.

#### Virus inoculation

The viral inoculation was performed as previously described [63,64]. Briefly, Huh-7 cells were grown in a 75 cm^2^ culture flask for 48 h to semi-confluence. The cells were then washed twice with serum-free medium, followed by inoculation with 500 μL serum obtained from HCV infected patients (5.8 × 10^8^ copies/mL) and 500 μL serum-free DMEM. After 180 min, DMEM medium containing serum was added. The cells were maintained at 37°C in 5% CO_2_.

#### siRNA transfection

Serum-inoculated Huh-7 cells (1 x10^5^) were plated into 24-well plates and transfected with siRNAs according to Zekri *et al*. [65]. Cells were transfected using HiPerFect transfection reagent (Qiagen) according to the manufacturer’s instructions. Briefly, the complex was prepared by diluting 2 μM HCV353 and HCV258 siRNAs stocks in DMEM without serum, adding HiPerFect, and incubating for 10 min at room temperature. The mixture was added drop-wise to the well (final siRNA concentration of 5 nM). AllStars Negative Control siRNA (Qiagen) was used as the reference standard.

#### Total RNA extraction

Total RNA was extracted at 24-h intervals for 3 consecutive days (24, 48, and 72 h post-transfection) using the RNeasy Mini kit (Qiagen), and was purified according to the manufacturer’s recommendations. The RNA contained in the two wells of a 24-well plate transfected with the same siRNA was pooled and stored at -80°C.

#### HCV RNA detection *via* quantitative real-time RT-PCR

HCV RNA concentrations were determined *via* quantitative real-time RT-PCR, as previously described by Zekri *et al*. 2009 [65]. Briefly, viral cDNA was generated by using 1 μg total RNA isolated using the OneStep RT-PCR kit (Qiagen) according to the manufacturer’s recommendations. HCV sequences were detected using the following forward 5' TGAGGAACTACTGTCTTCACG 3' and reverse 5' ACTCGCAAGCACCCTATCAGG 3' primer set. The GAPDH gene (GenBank accession no. NMX002046) was selected as a normalization control and detected using a forward 5' GAAGGTGAAGGTCGGAGTC 3' and reverse 5’ GAAGATGGTGATGGGATTTC 3' primer set.

### Data Analysis

All values generated *via* luciferase-, GFP-, and qRT-PCR-based read-outs were expressed as mean ± standard error (±SEM) results. Comparison of the antiviral potency of different siRNAs was made using a two-tailed Student’s *t*-test. A *p*-value of *p*<0.05 (*) was considered to indicate a statistically significant result. P-values of *p*<0.01 (**) and *p*<0.001 (***) were considered to indicate highly significant and extremely significant results, respectively.

## Results

### HCV Genome Analysis, *In Silico* siRNA Design and Selection

After performing individual whole-genome alignments for each HCV genotype, conserved RNAi target regions were identified. The design and scoring of siRNAs targeting specific regions of the HCV RNA genome was performed using siVirus, i-Score, and Convenient Application Program for siRNA Design (CAPSID) software [34,43,44]. siRNAs targeting regions that were not conserved between the main HCV genotypes were excluded. The final siRNAs were those that targeted highly conserved regions across all HCV genotypes (Table 1 and S1 Table). siRNAs with off-target matches in the human RefSeq mRNA database were not considered. This specificity check was scrutinized using a Smith-Waterman-like search that uses more rigorous search criteria (dynamic programming) compared with those of the typical BLAST algorithm [53]. Fig 2 presents an outline of the strategy used to filter *in silico*-designed siRNAs according to the highest scores, thermodynamic parameters, and off-target considerations.

Following this procedure, 322 siRNAs were designed, targeting conserved regions in the HCV 5′NTR (data not shown). After off-target exclusion prediction, siRNAs expected to trigger minimal miRNA-like off-target effects, causing the down-regulation of targeted genes, were rejected. After these multiple steps used to improve target-specific RNAi efficacy, the resulting siRNA candidates were further narrowed down to the HCV353 siRNA (Table 1). This siRNA was predicted to be the most potent and highly HCV-specific candidate, based on good target accessibility and lack of major off-target effects. HCV353 siRNA targets highly conserved sequences in stem loop IV of the HCV IRES. These sequences are highly conserved between all of the HCV genotypes (i.e., 1 to 6). Another less favorable siRNA (HCV258; Table 1) targeting a region in the 5′NTR stem loop IIId, which is only conserved in up to 89.5% of all HCV genotypes, was also chosen to be experimentally validated in cell culture. This reduced homology was exhibited by a mismatch in the IRES of genotype 2. Due to the superiority of HCV353 siRNA in terms of its competent thermodynamic features, high conservation, and predicted lower thermodynamics accessibility, reduced antiviral efficacy was expected for HCV258 siRNA. HCV258 targets an RNA region that is only conserved to 89.5% and has mismatches in the JFH-1 seed sequence, and which, therefore, did not pass the target accessibility criteria using RNAxs tool [58]. Both *in silico*-designed siRNAs were compared with previously described siRNAs that target the HCV 5′NTR and that were used as references (HCV321 and HCV360) [66,67]. Fig 1 presents the results for an alignment of HCV258 and HCV353 and the two reference siRNAs to the HCV 5′NTR. Binding to the target sequence was visualized using this comparison. In summary, we *in silico*-designed HCV-specific siRNAs predicted to be highly efficacious, with a high barrier to resistance and with a low probability of off-target effects.

### Experimental Validation of *In Silico*-Designed siRNAs in HCV Replicon Cell Lines

#### Dose-dependent antiviral effects of IRES-specific siRNAs on the replication of subgenomic HCV replicons expressing NS5A-GFP fusion protein

To validate the predicted potency and specificity, we performed knockdown experiments with the two selected *in silico*-designed siRNAs and compared them to previously described siRNAs targeting the 5′NTR or adjacent core sequences within the IRES. For the target cells, we used subgenomic HCV replicon cell lines expressing an NS5A-GFP fusion protein. Insertion of the GFP-encoding sequence in HCV NS5A cells allowed the non-invasive monitoring of HCV RNA replication dynamics because GFP fluorescence intensity is proportional to viral replication. First, we devised knockdown experiments in Huh-7 cells, which are widely used to study HCV, and replicated either genotype 1b (Con-1) or genotype 2a (JFH-1) subgenomic replicons expressing an NS5A-GFP fusion protein. To corroborate the results using Huh-7 cells, we also used the hepatoblastoma cell line HuH6 and replicated JFH-1 replicons.

**Fig 3:**
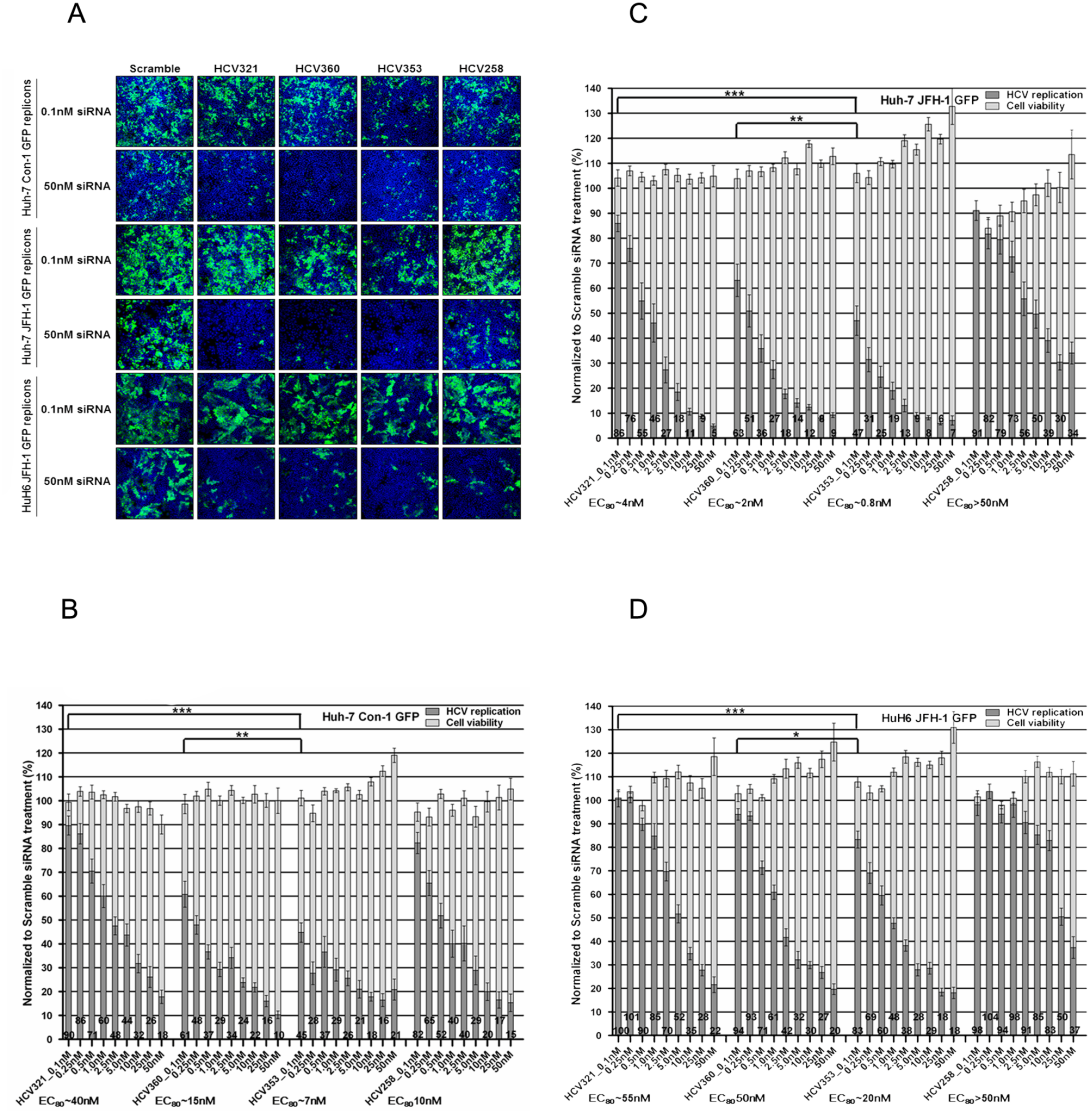
Dose-dependent antiviral effects of HCV IRES-specific siRNAs on the replication of subgenomic HCV replicons expressing a NS5A-GFP fusion protein. (A) Representative confocal images of GFP replicon cells after transfection with HCV-specific siRNAs. Replicon cells were PFA-fixed, and stained with Hoechst 33432 stain. Images of 0.1 and 50 nM siRNA transfections at 72 h post-transfection (p.t.) are shown (ImageXpress Ultra: 20x magnification). Overlaid images: cell nuclei (blue) and HCV RNA replicating cells (green). GFP replicon cells: (B) Huh-7 Con1, (C) Huh-7 JFH-1, and (D) HuH6 JFH-1 replicon cells were plated and transfected with 0.1–50 nM siRNAs targeting the viral genome and a scrambled control. At 72 h p.t., confocal images of GFP replicon cells were acquired and analyzed using an ImageXpress Ultra microscope and MetaXpress software, respectively. Results were normalized to the level of GFP-positive cells of the scrambled siRNA transfection control, which was set to 100%. The threshold of GFP was defined by treatment of replicon cells with an HCV replication inhibitor, which was set to 100% inhibition (data not shown). Fluorescence intensity above this threshold was considered to indicate active HCV replication. Data are presented as the mean ± SEM values for four wells measured in quadruplicate in two independent experiments (N = 32). Black columns indicate HCV RNA replication (GFP-positive cells); grey columns indicate cell viability (total cell number of Hoechst 33432 stained cell nuclei). The numbers on the bars indicate the residual percentage of GFP-positive cells. Asterisks indicate that the mean values are significantly different between samples (*p<0.05; **p<0.01; ***p<0.001).

At 20 h after cell plating, siRNAs were transfected into cells at defined concentrations (0, 0.1, 0.25, 0.5, 1, 5, 10, 25, or 50 nM). HCV RNA replication and cytotoxicity were determined 72 h post-transfection (p.t.) using dose-response curve analysis. The efficient concentration required to inhibit 80% of viral replication (EC_80_) was calculated. The previously described siRNAs HCV321 and HCV360 targeting the HCV 5′NTR were used as positive controls. A scrambled siRNA that was designed to target neither cellular RNAs (off-target effects) nor the HCV RNA genome served as the negative control [66,67]. Viral replication capacity and cell viability for each individual siRNA were determined as described in the Materials and Methods section. The data were normalized to those of the scrambled siRNA transfection control. Briefly, experiments were conducted in duplicate in 384-well microplates. At 72 h p.t., four digital images per well were taken and the total cell numbers were determined. Cells expressing GFP were identified by measuring fluorescence intensity on a single cell basis. Representative confocal images used to visualize HCV RNA replication inhibition in subgenomic replicon cells expressing an NS5A-GFP fusion protein after siRNA transfection are presented in Fig 3A. The digital images correspond to the results presented in Fig 3B, 3C and 3D. Transfection of all tested siRNAs targeting the 5′NTR led to the dose-dependent inhibition of HCV genotype 1 (Con-1) RNA replication in Huh-7 cells, without a reduction in cell viability. The slopes of inhibition were comparable between HCV321 (EC_80_ ~40 nM) and HCV258 (EC_80_ ~10nM) and between HCV360 and HCV353 siRNAs at 72 h after transfection. Notably, viral inhibition was more efficient after transfection with HCV360 and HCV353 siRNAs, with EC_80_ values of approximately 15 nM and 7 nM, respectively. We used a two-tailed Student’s *t*-test and calculated *p-*values to reveal whether our *in silico*-designed HCV353 siRNA had a statistically significant higher antiviral potency than that of the reference siRNAs, HCV321 and HCV360. For this purpose, we selected the lowest siRNA concentration (0.1 nM) because this concentration is suitable to determine even minor, but significant, differences in viral replication inhibition by individual siRNAs. The inhibition of genotype 1 viral replication by HCV353 siRNA was 1.4-fold (*p* = 0.0084) and 5.3-fold (*p*<0.0001) stronger, compared with that by HCV360 and HCV321 siRNAs, respectively.

Using the identical experimental setup as described for genotype 1 GFP-expressing replicons, the replication of genotype 2 (JFH-1) subgenomes in Huh-7 cells was determined after transfection of siRNAs targeting the 5′NTR (Fig 3C). Consistent with the observation in Huh-7 cells replicating Con-1, a dose-dependent antiviral effect was revealed. HCV321 (EC_80_ ~4 nM), HCV360 (EC_80_ ~2 nM), and HCV353 (EC_80_ ~0.8 nM) siRNAs showed stronger inhibitory effects than did HCV258 siRNA (EC_80_ >50 nM). At a concentration of 0.1 nM, the inhibition of genotype 2 viral replication was 1.4-fold and 3.8-fold stronger with HCV353 siRNA than with the HCV360 and HCV321 siRNAs; these results were highly (*p* = 0.001), and extremely (*p*<0.0001), significant, respectively. In HCV genotype 1 and 2 Huh-7 replicon cells, transfection of HCV353 siRNA led to an increase in total cell number in a dose-dependent manner.

To corroborate our results in the hepatoma cell line Huh-7, we used a different cell line to exclude cell line-specific effects. We performed transient knockdown experiments in the human hepatoblastoma cell line HuH6 [59] replicating genotype 2 (JFH-1) subgenomes. The experimental setup and data analysis were comparable to the experiments using Huh-7 cells and are described in the Materials and Methods section. Consistent with the result with Huh-7 cells replicating JFH-1, a dose-dependent antiviral effect was observed in HuH6 replicon cells (Fig 3D). The EC_80_ values were at least 5-fold higher than in the Huh-7 replicon cell lines (HCV321 EC_80_ ~55 nM, HCV360 EC_80_ 50 nM, HCV353 EC_80_ ~20 nM, and HCV258 EC_80_ >50 nM). To determine whether the results were statistically significant, *p-values* were calculated for the lowest siRNA concentration. Inhibition of genotype 2 viral replication in HuH6 cells was 2.8-fold stronger with HCV353 siRNA than with HCV360 (*p* = 0.036). However, HCV321 siRNA did not inhibit viral replication at 0.1 nM (*p* = 0.0005). Similar to the results obtained with HCV genotype 1 and 2 Huh-7 replicon cells, transfection with siRNA HCV353 led to an increase in total cell number in a dose-dependent manner. This effect also occurred after the transfection of siRNA HCV360 into HuH6 replicon cells. Taken together, these results indicated that our *in silico*-designed siRNA HCV353 was extremely potent and specifically interfered with HCV RNA replication, irrespective of HCV genotype and cell origin. We ranked the antiviral efficacies of the various siRNAs based on their EC_80_ values and obtained an order of HCV353 > HCV360 > HCV 321 > HCV258. However, in Huh-7 Con-1 GFP replicon cells, the ranking of the HCV 321 and HCV258 siRNAs was reversed; the reason for this result remains unknown.

#### Dose-dependent antiviral effects of IRES-specific siRNAs on the replication of subgenomic HCV replicons expressing *Firefly* luciferase

**Fig 4:**
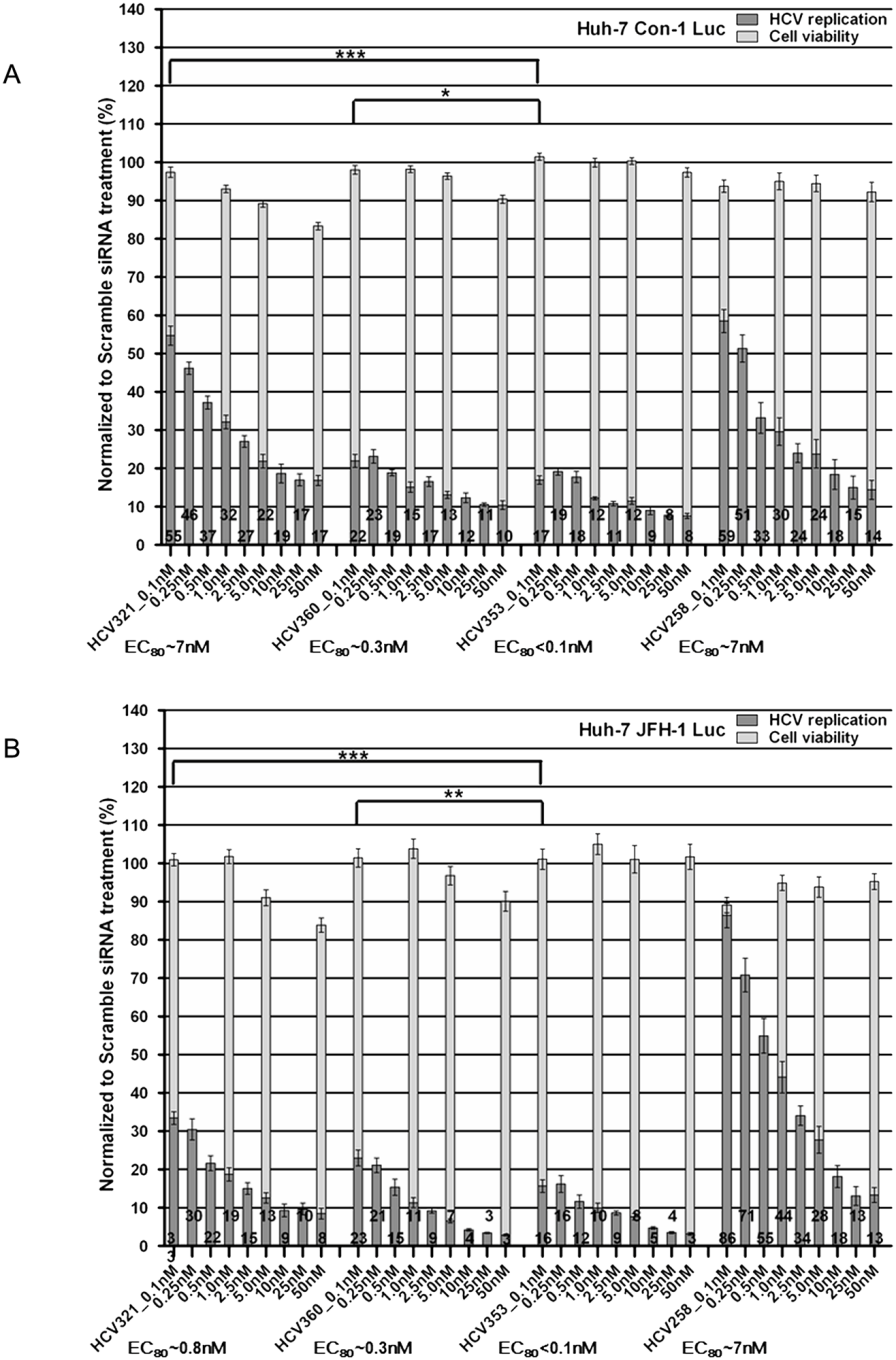
Dose-dependent antiviral effects of HCV IRES-specific siRNAs on the replication of subgenomic HCV replicons expressing *Firefly* luciferase. Luciferase replicon cells: (A) Huh-7 Con1 and (B) Huh-7 JFH-1 replicon cells were plated and transfected with 0.1–50 nM siRNAs. At 72 h post-transfection (p.t.), cells were harvested for luciferase activity measurement. *Firefly* luciferase activity (HCV RNA replication) and cellular ATP content (cell viability) of the scrambled siRNA transfection were set to 100% and used to normalize the relative luciferase activities of all other siRNA-transfected cells. Data are presented as the mean ± SEM values of four wells measured in duplicate in two independent experiments (N = 24). Black columns indicate HCV RNA replication; grey columns indicate cell viability. The numbers on the bars indicate the residual percentage of luciferase activity. Asterisks indicate that the mean values are significantly different between samples (*p<0.05; **p<0.01; ***p<0.001).

To corroborate the results obtained by phenotypic analysis of HCV replication, we employed Huh-7 cells containing subgenomic replicons encoding the *Firefly* luciferase gene [68]. This stable cell line is widely used to evaluate HCV replication inhibitors. Similar to the experimental setup described for replicon cells expressing an HCV NS5A-GFP fusion protein, cells were lysed at 72 h after siRNA transfection. Measurements of total relative light units and cellular ATP levels were used to determine luciferase activity and cytotoxicity, respectively. Transfection of all siRNAs targeting the HCV 5’NTR led to dose-dependent inhibition of HCV genotype 1 (Con-1) and genotype 2 (JFH-1) RNA replication, without affecting cell viability (Fig 4A and 4B). Dose-response curve analysis revealed that the HCV360 (EC_80_ ~0.3 nM) and HCV353 (EC_80_ <0.1 nM) siRNAs inhibited viral replication considerably more efficiently than did the HCV321 and HCV258 siRNAs (EC_80_ for both ~7 nM). This result was consistent with the results of the phenotypic analysis. The EC_80_ values calculated for individual siRNAs in genotype 1 and 2 replicons were nearly identical, except that HCV321 siRNA had a nearly 10-fold lower activity in Con-1 replicating cells. This result was also consistent with the phenotypic analysis result.

Based on *p*-values calculated as described earlier in the Materials and Methods section, we found that our *in silico*-designed HCV353 siRNA had a statistically significant higher potency in inhibiting HCV RNA replication, compared with the HCV321 and HCV360 siRNAs. In summary, these results were consistent with the results obtained using GFP-expressing replicon cells and confirm the ranking of siRNAs according to the antiviral efficacy obtained using phenotypic readout. However, we found that there was a general reduction in EC_80_ values when *Firefly* luciferase-expressing HCV replicons were used. This reduction was very likely due to differences in the half-lives of NS5A-GFP and luciferase. These results also indicated that our phenotypic assay is robust, highly reproducible, and suitable for determination of the efficacy of HCV RNA replication inhibitors.

#### Time-dependent antiviral effects of IRES-specific siRNAs on the replication of subgenomic HCV replicons expressing *Firefly* luciferase

**Fig 5:**
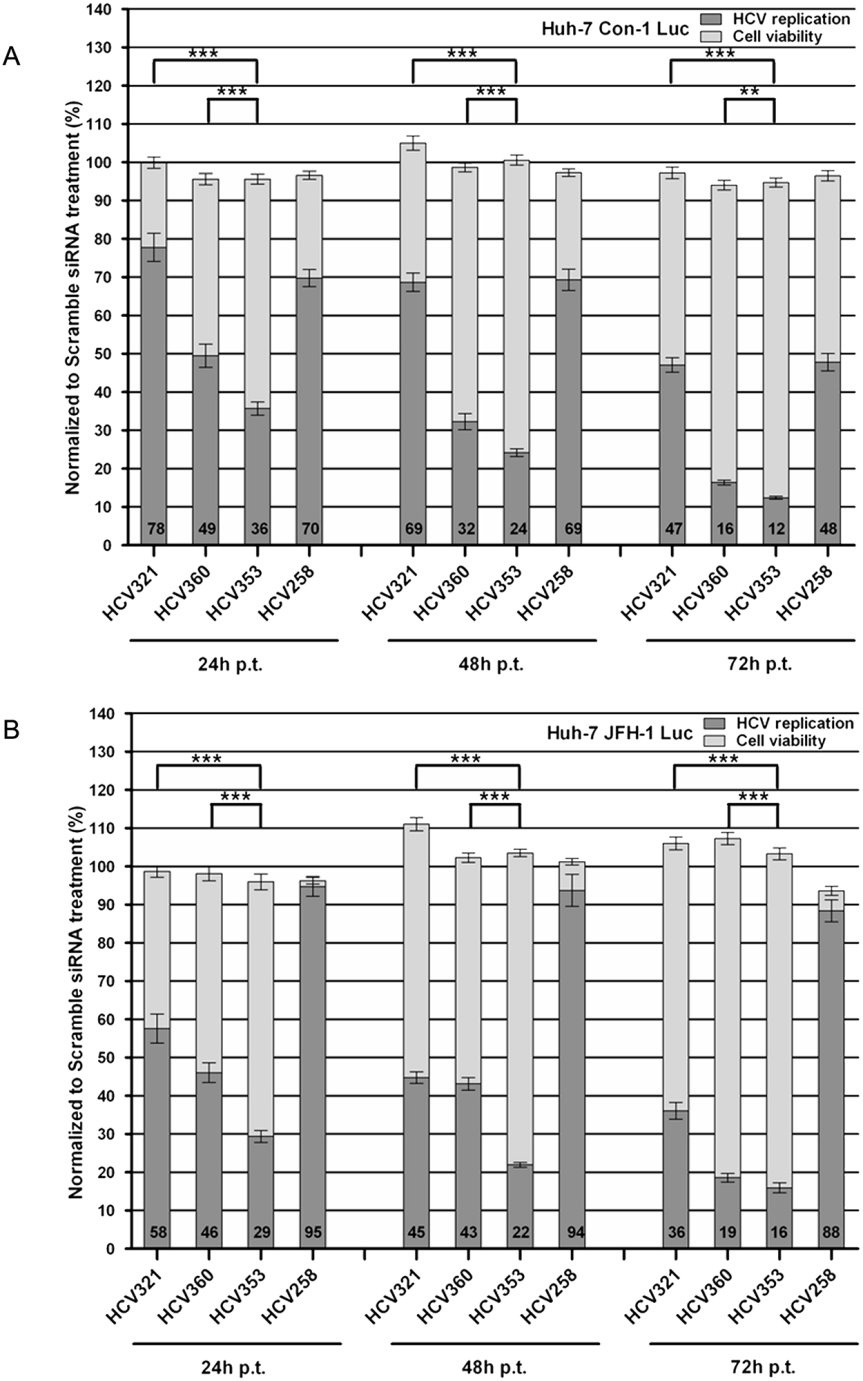
Time-dependent antiviral effects of HCV IRES-specific siRNAs on the replication of subgenomic HCV replicons expressing *Firefly* luciferase. Luciferase replicon cells: (A) Huh-7 Con1 and (B) Huh-7 JFH-1 replicon cells were plated and transfected with 0.1 nM siRNAs and analyzed as described in Fig 4. At the indicated time points (24, 48, 72 h post-transfection), cells were lysed and luciferase activities were measured. Relative luciferase activities were normalized to that of the scrambled siRNA transfection control.

After the precise determination of the dose-dependent antiviral effects of HCV 5′NTR targeting siRNAs, we devised time-course experiments to further investigate differences between the antiviral potencies of our *in silico*-designed siRNAs. siRNAs were transfected at a concentration of 0.1 nM into Huh-7 luciferase replicon cell lines. At 24, 48, and 72 h p.t., the cells were lysed and luciferase activity and cytotoxicity were determined as described. Silencing effects were detectable as early as 24 h after transfection. The greatest reduction in viral replication occurred within the first 24 h. Even at this early time point, HCV353 siRNA inhibited genotype 1 and genotype 2 replication up to 64% and 71%, respectively (Fig 5A and 5B). At 48 and 72h p.t., replication was further inhibited up to approximately 90% by siRNA HCV353. The reference siRNA, HCV360, which was the second most potent siRNA in our study, exhibited very statistically significant lower antiviral potencies at all time points, as indicated by the *p*-values. Reduced cell viability, which might interfere with HCV replication, was not observed at any time point. Consistent with the results of previous experiments, the HCV321 and HCV258 siRNAs shared comparable antiviral potencies in inhibiting genotype 1 replicons. HCV258 siRNA inhibited HCV genotype 2 replication, but only poorly. Taken together, these results corroborate the results obtained by our dose-response analysis in diverse subgenomic HCV replicon cell lines. At all time points and in both time-course experiments with genotype 1 and 2 replicon cell lines, the previously observed ranking of antiviral efficacy was confirmed (HCV353 > HCV360 > HCV 321 > HCV258). The outstanding antiviral potency/efficacy and selectivity of our *in silico*-designed HCV353 siRNA was statistically significant, compared with the reference siRNAs.

### Time-Dependent Antiviral Effects of IRES-Specific siRNAs on Patient-Derived HCV Genotype 4 Isolates

**Fig 6:**
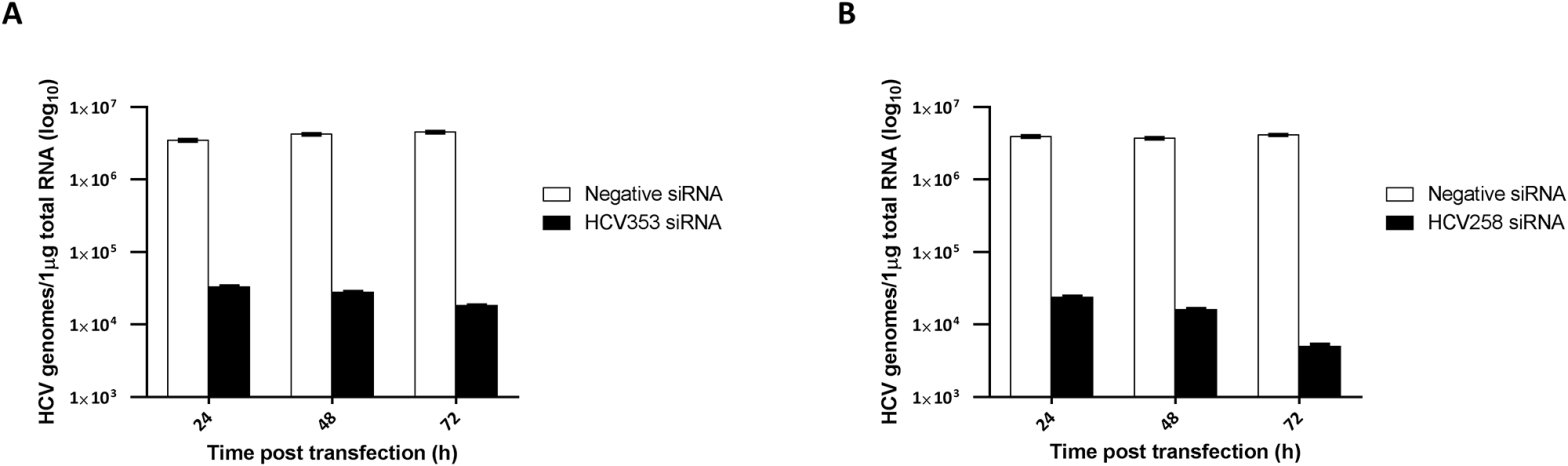
Time-dependent antiviral effects of HCV IRES-specific siRNAs on patient-derived HCV genotype 4 isolates. Huh-7 cells infected with patient-derived genotype 4 isolates, transfected with HCV353, HCV258, and negative control siRNAs were harvested at the indicated time points (24, 48, 72 h post-transfection) and HCV genomes determined using qRT-PCR analysis. Black and white columns indicate HCV-specific siRNA and negative control siRNAs, respectively. X-axes and y-axes depict the time post-transfection and HCV genomes, respectively. (A) HCV353 and (B) HCV258 siRNA inhibition of HCV genotype 4. Data are presented as the mean ± SEM values of two wells measured in triplicate.

To corroborate the results using stably HCV-replicating replicon cell lines, we performed experiments using patient-derived HCV genotype 4 isolates. Genotype 4 is the most prevalent genotype in Egypt. The objective of these experiments was to determine whether our *in silico*-designed siRNAs could inhibit authentic viruses [1,63]. Naïve Huh-7 cells were inoculated with serum from viremic patients according to Awady *et al*. 2006 [64]. Briefly, Huh-7 cells were grown for 48 h to semi-confluence in 75 cm^2^ culture flasks, washed with serum-free medium, and then inoculated with 500 μL serum derived from HCV infected patients (5.8 × 10^8^ HCV RNA copies/mL). The cells were harvested at 72 h post infection, plated into 24-well plates, and transfected with HCV258 or HCV353 siRNA at a final concentration of 5 nM. The cells were then harvested at various time points post-transfection (24, 48, and 72 h), the total RNA was isolated, and HCV RNA was assessed quantitatively *via* real-time PCR. The results indicated that the HCV353 and HCV258 siRNAs significantly inhibited HCV genotype 4 replication in a time-dependent manner (Fig 6A and 6B). Efficacy of HCV inhibition with both siRNAs was comparable at 24 and 48 h p.t. At 72 h p.t. the HCV353 and HCV258 siRNAs inhibited genotype 4 replication 248- and 824-fold, respectively, compared with the negative siRNA control, which did not interfere with HCV RNA replication. In summary, these results revealed the strong antiviral potency of our *in silico*-designed siRNAs on authentic patient-derived HCV.

### Curing of HCV Replicons with IRES-Specific siRNAs

**Fig 7:**
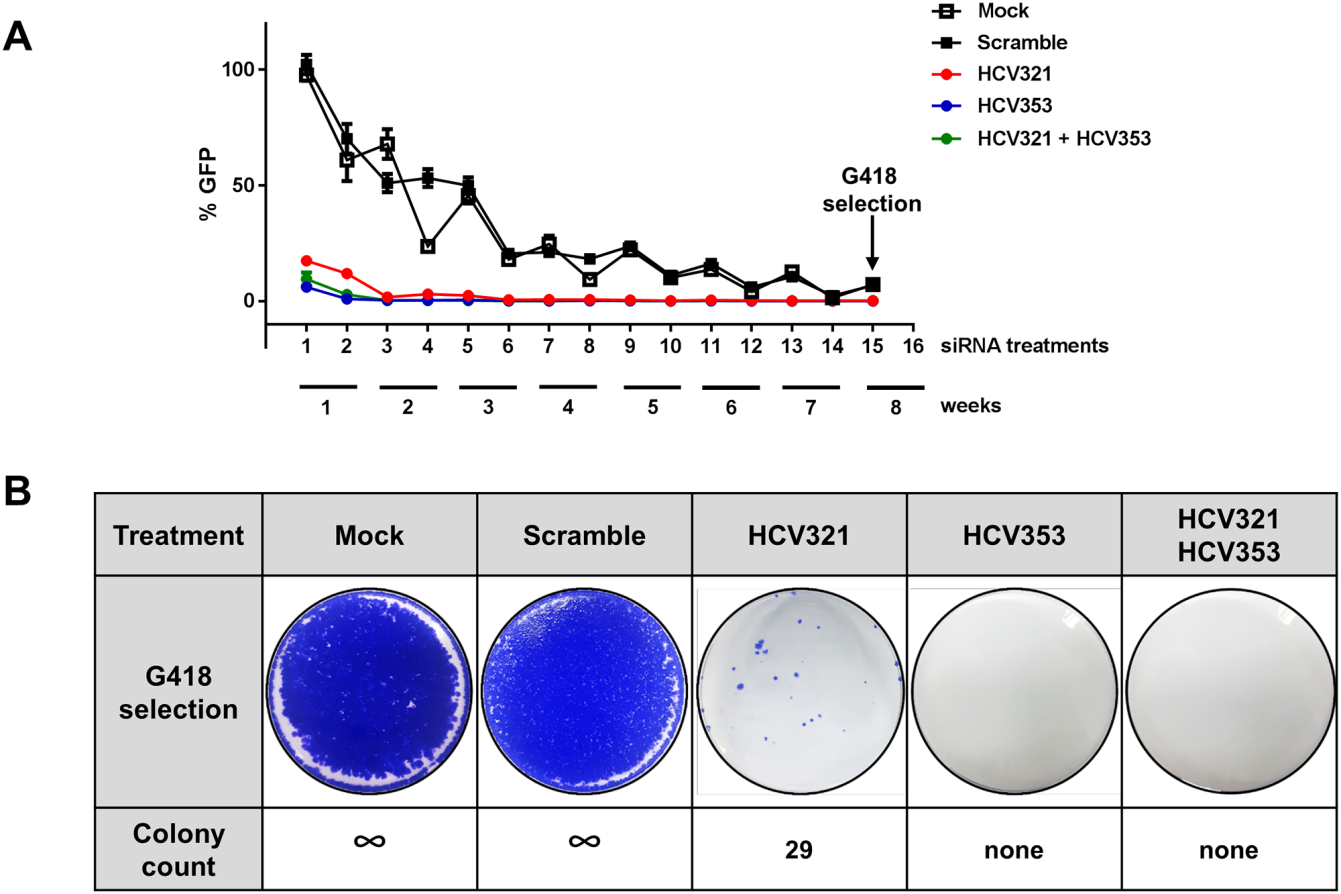
Curing of HCV replicon cells with IRES-specific siRNAs. (A) Subgenomic HCV JFH-1 replicon cells expressing an NS5A-GFP fusion protein were treated in the absence of G418 twice per week with 50 nM of siRNAs, either individually (scramble, HCV321 or HCV353) or in combination (50 nM HCV321 and 50 nM HCV353) for 8 weeks as depicted. HCV RNA replication was determined twice per week using GFP expression. (B) siRNA transfection was discontinued after 8 weeks, replicon cells were treated with 500 μg/mL G418 for 4 weeks, and resistant cell clones were stained using crystal violet.

To determine whether our siRNAs were less likely to lead to viral escape, we treated HCV replicon cells biweekly with 50 nM of siRNAs either individually (scramble, HCV321 and HCV353) or and in combination (HCV321 + HCV353) for 2 months without G418 selection. HCV RNA replication was determined biweekly by monitoring NS5A-GFP expression (Fig 7A). At 72 h after the first treatment, there was an 80% reduction using HCV321, a 90% reduction using HCV321 + HCV353, and a >90% reduction using HCV353. After three treatments, all siRNAs inhibited HCV replication by >99%. Over time, the mock and scramble controls reduced HCV RNA replication due to the lack of G418 selection pressure. siRNA transfection was discontinued after 8 weeks of treatment, the replicon cells were treated with 500 μg/mL G418 for 3 weeks, the resistant cell clones were visualized using crystal violet staining, and the G418-resistant clones were counted. The mock and scramble control results indicated that there were uncountable high amounts of G148-resistant clones, whereas transfection of HCV321 siRNA resulted in 29 colonies (Fig 7B). However, treatment of replicon cells with HCV353, alone or in combination with HCV321, did not lead to the generation of viral escape variants, which indicated the presence of a high genetic barrier to resistance of our *in silico*-designed siRNA.

## Discussion

Exceptional advances have been made in biological research in recent years. RNAi technology is one of the major discoveries that is changing the scientific landscape. RNAi is a highly specific post-transcriptional regulatory pathway that results in the silencing of gene function. Investigators have worked to translate this basic scientific finding into therapeutic interventions for patients. However, the bench-to-bedside approach has been accompanied by technical issues that hamper the development of therapeutic RNAi technology. Stability issues, off-target effects, immunostimulatory activities, and delivery liabilities all contributed to slowing down progress. In recent years, researchers have successfully surmounted these obstacles and improved crucial properties such as bioavailability, safety, and delivery strategies. RNAi-based drugs have been approved by the FDA. Significant investment and collaboration with mid-sized companies by large pharmaceutical companies has indicated interest in RNAi technology.

With the recent approval of HCV inhibitors, significant progress has been made in the development of novel HCV-specific DAAs. For decades, chronically infected patients relied on a virus-non-specific combinatorial therapy of PEG-IFNα and ribavirin with low SVRs and adverse effects. However, despite the development of DAAs, most patients worldwide are still treated with IFNα. There are many reasons for this continued reliance on IFNα. Patients have a wait-and-see attitude about the first-generation protease inhibitors boceprevir and telaprevir because therapy is accompanied by severe side effects, has a low resistance barrier, and follows an inconvenient administration regime. The recently approved drugs simeprevir and sofosbuvir have been only slowly introduced into the clinics. The high economic burden of approximately USD 100,000 for 12 weeks of therapy (e.g., sofosbuvir) will also limit access to therapy. Because the viral polymerase (NS5B) lacks a proofreading function, antiviral efficacy is challenged by a swarm of genetically distinct but closely related variants (“quasispecies”). Therefore, antiviral therapy will potentially lead to a complex mixture of drug resistance viruses, and even the new drugs with potential will encounter development of resistance [69]. These unmet medical needs encouraged us to investigate alternatives, to identify more affordable, safer, and potent drugs for HCV treatment that have a high genetic barrier to drug resistance and high pan-genotypic activity.

The highly structured IRES, 5′ and 3′NTRs, and double-stranded RNA replicative intermediates of HCV are attractive targets for RNAi. Because this RNA virus replicates only within the cytoplasm, RNAi therapy has potential to cure chronically infected patients. By targeting RNA regions that are highly conserved among different HCV genotypes and analyzing genome alignments, we selected the viral IRES element and adjacent core sequences that were good candidates for siRNA design [70]. We designed HCV-specific siRNAs using an automated RNAi design tool, which was capable of designing specific siRNAs while accounting for multiple transcript filtration and target accessibility, and off-target filtration evaluation [39]. To prevent potential adverse effects, off-target analysis was performed to avoid non-specific targeting of cellular mRNAs and was combined with an automated workflow that was able to reproduce the results. This analysis thus serves as a proof of concept for the MysiRNA-Designer, which performs similar steps in an automated fashion. The first goal of this study was to design potent siRNAs *in silico* targeting highly conserved regions in the HCV IRES and adjacent core sequences. Our paradigm was to use, integrate, and consider all crucial factors for efficient siRNA design and selection. Using a novel procedure, we filtered siRNAs based on merged scores exceeding thresholds, target accessibility, and off-target analysis. Our second goal was to experimentally validate the predicted antiviral potency of selected siRNAs in the replicon system by using different HCV genotypes, diverse reporter genes for viral replication, and human liver cell lines of different origin.

Here we present a description of a novel methodology used to target highly mutating RNA viruses; we applied this methodology to HCV. The methodology aimed to design optimal siRNAs using careful analysis of the target sequence, structure, and space constraints. A multi-step off-target analysis using both near-perfect complementarity and seed-region matching was applied to target highly conserved areas in the IRES region. Target accessibility was then evaluated by combining computational and practical methods. The improved target accessibility and thermodynamic considerations contributed significantly to high siRNA silencing efficacy. Different computational techniques were used to predict high single-stranded target region probabilities. These techniques included analysis of the siRNA thermodynamic profiles to ensure differential-end instability and unstructured folding (non-stable siRNA folding energies). Increased specificity towards the targeted virus was achieved by improvements in off-target scoring schemes [71] and searching within seed matches to the 3′NTR of human mRNAs [72] (Fig 2). The latter used two-phase off-target screening that eliminated the nearly perfect matches in the first phase; seed-region matches with 3′NTRs of human mRNAs were eliminated in the second phase.

The outputs of our extensive bioinformatics predictions were siRNAs targeting highly conserved, highly accessible, and biologically critical regions of the viral RNA genome, with predicted high antiviral potency across all HCV genotypes. This procedure represents a novel methodology for siRNA design and cell-based validation that could be applied to other pathogenic viruses in the quest for new viral interventions. An in silico screening of human miRNAs targetting the Hepatitis c virus was also performed before[73].

In this study, two *in silico*-designed siRNAs were selected and experimentally validated in the HCV replicon system. To precisely monitor antiviral effects, we compared our *in silico*-designed siRNAs to previously described siRNAs in a time- and dose-dependent manner. We used cell lines of different origins (hepatoma and hepatoblastoma), different HCV genotypes (1, 2, and 4), different reporters for HCV RNA replication (GFP and *Firefly* luciferase), and patient-derived HCV isolates. Using this strategy, we ruled out reporter and cell line artifacts and HCV genotype specificity. Subgenomic HCV replicon cell lines expressing *Firefly* luciferase or GFP were transiently transfected with siRNAs, and bioluminescence or fluorescence intensity, respectively, was measured at the end of each experiment. The EC_80_ values of individual siRNAs were calculated and ranked to compare the antiviral potency of all tested siRNAs. Dose-response curve analysis was used to rank the antiviral potencies of the individual siRNAs. The rank order was corroborated using time-dependent experiments (HCV353 > HCV360 > HCV 321 > HCV258). Irrespective of differences in tools and the experimental setup used to assess HCV RNA replication inhibition, the outstanding antiviral efficacy and selectivity of our *in silico*-designed HCV353 siRNA was statistically significant, compared with the reference siRNAs. We also extrapolated the EC_50_ values of HCV353 siRNA-transfected luciferase-expressing replicon cell lines within a low two-digit picomolar range. The HCV353 and HCV360 siRNA seed sequences overlapped with 12 identical nucleotides in the Con1 and JFH-1 genomes, which might explain the similar antiviral potencies. Sequences proximal to the start codon for protein synthesis are favorable for the efficient inhibition of HCV RNA translation, which suggests that this region is an excellent target due to its good accessibility to RNAi [74]. The higher antiviral potency of HCV353, compared with HCV258 siRNA was expected because it had superior thermodynamic characteristics, and because there were two nucleotide mismatches of HCV258 siRNA in the seed region within the JFH-1 IRES.

By comparing the Huh-7 and HuH6 JFH-1 GFP replicon cell lines originating from hepatoma and hepatoblastoma, respectively, we found >10-fold higher EC_80_ values in HuH6 cells. Results from colony formation and transient transfection experiments indicate that this cell line is less permissive to HCV RNA replication [16]. The higher EC_80_ values in HuH6 cells may reflect differences in transfection efficiencies, or lower RISC activity. The GFP fluorescence intensity, which is proportional to viral RNA replication, is significantly lower in HuH6 cells (data not shown). Additionally, HCV-specific siRNAs may interfere mainly with newly-formed replication complexes (RCs), which remain stable for up to 18 h, rather than affecting the activity of pre-existing ones [75,76]. The hypothesis of a lower RC turnover rate in HuH6 cells is consistent with the observation of delayed antiviral activity of non-nucleoside inhibitors (NNIs) in HuH6 replicon cells [16]. Our hypothesis is also supported by the observation that NNIs fail to inhibit HCV RNA synthesis by native replicase complexes isolated from replicon cells [77]. A shift to higher EC_80_ values in HuH6 replicon cells did not occur using HCV-specific NS5A and NNIs. This result suggested that these antivirals had access to pre-existing RCs (data not shown). It is also unlikely that RISCs, 5′ phosphomonoester-producing RNA endonucleases, with a size of approximately 160 kDa, are capable of entering pre-existing RCs; even proteinase K (29 kDa) and S7 nuclease (17 kDa) cannot enter crude RCs [78].

With the generation of HCV replicons expressing the *Firefly* luciferase gene as a marker of viral RNA replication, it has become possible to very conveniently measure luciferase activity. This actively is indirectly proportional to viral replication and translation. The identification of a region in the NS5A domain III that tolerates insertion of the GFP coding sequence enables non-invasive visualization of HCV RNA replication dynamics in live or fixed cells. The amount of NS5A-GFP fusion protein expression is directly proportional to actual HCV RNA replication [79].

By phenotypically visualizing NS5A, the content obtained from each individual experiment was increased because we did not investigate only a reporter whose half-life does not correlate to the half-life of viral proteins. This type of cellular imaging provides other valuable *in vitro* information (e.g., cell morphology, protein localization, cytotoxicity), which may useful for understanding other pathological conditions and cellular events. By analyzing images from our dose-response experiments, we noticed that the EC_80_ values were consistently higher in the GFP replicon cell lines. It is tempting to speculate that the higher half-life of the NS5A-GFP fusion protein (approximately 16 h as measured by pulse-chase experiments using HCV replicon cells *vs*. 3 h for the *Firefly* luciferase protein) might be responsible for this result [80,81]. However, by using the small molecule inhibitors telaprevir or sofosbuvir, no significant differences in EC_50_ values were observed after 72 h p.t. (data not shown). This result indicated that due to the short half-life of the *Firefly* luciferase, it was more difficult to reveal differences in RC turnover rates after 72 h post siRNA transfection. Additionally, by quantifying cell nuclei, which was a marker for cytotoxicity, we could detect an increase in number of cell nuclei in efficiently HCV-inhibited replicon cell lines. This effect was more pronounced in HCV353 siRNA-transfected replicon cells, which suggested that cellular resources were handed back to the host. This phenomenon was also observed after the treatment of HCV replicons or infected cells with small molecule inhibitors (data not shown) and was corroborated using commercial reagents to measure cytotoxicity.

Using different HCV genotypes and viral reporters, we found that EC_80_ values were significantly higher in GFP replicon cells, especially with Con-1 replicons. This phenomenon was not apparent in luciferase-expressing replicon cells, presumably due to a lower half-life of the reporter, which did not allow the precise monitoring of HCV replication. The GFP fluorescence intensity is directly proportional to viral RNA replication, which is significantly greater in JFH-1 replicating cells. This observation corroborates our hypothesis that HCV RC turnover rates are affected by the viral replication efficacy and permissiveness of the cell. The reduced replication efficacy of genotype 1 replicons in HuH6 cells, which are less permissive to HCV RNA replication, reduced the RC turnover rate. However, the steps involved in the formation of the membranous web appear to be more complex. The interactions between viral and cellular proteins and the turnover rates of RCs in different HCV genotypes remain largely unknown. Differences in antiviral efficacy were apparent in NS5A-GFP, but were surprisingly minor in *Firefly* luciferase-expressing Huh-7 replicon cell lines. This result indicates that the sensitivity was lower luciferase-expressing cells for determining antiviral efficacies and suggests that depending on the scientific question, differences in viral RNA replication inhibition can be more precisely monitored using phenotypic assessment.

HCV replicon cell lines are excellent tools for convenient study of viral replication, however only the infectious HCV cell culture system supports study of the entire viral life-cycle. In order to determine whether authentic patient-derived HCV isolates could be inhibited by our *in silico*-designed siRNAs, we performed experiments using genotype 4 isolates derived from Egyptian patients. Nearly 15% of the Egyptian population, which corresponds to 12 million patients, is chronically infected with this genotype [82]. Infection of naïve Huh-7 cells with patient-derived HCV genotype 4 isolates and transient transfection with the siRNAs HCV258 and HCV353 revealed that the antiviral efficacy of both siRNAs was similar; both aligned 100% to the genotype 4 RNA sequences. Both siRNAs also led to a similar HCV RNA reduction at 24 and 48 h p.t. However, at 72 h p.t., HCV258 siRNA resulted in a 3-fold stronger HCV inhibition compared with HCV353. This result occurred in three independent experiments (data not shown) and remains to be elucidated. In summary, we validated our *in silico*-designed siRNAs by obtaining strong antiviral effects on authentic patient-derived HCV and thereby corroborated our results with the HCV genotype 1 and 2 replicon cell lines. Finally, no viral escape occurred when we passaged replicon cells in the presence of HCV353 alone or in combination with HCV321. This result indicated that HCV353 has a high genetic barrier to resistance by targeting a highly conserved and crucial region in the HCV RNA genome. A previous study using an siRNA targeting the viral genome at position HCV359, which is very close to our target sequence, revealed that 10 treatments with a single siRNA at a 0.1 nM concentration per treatment over a period of 60 days does not result in clearance [83]. However, the combination of two siRNAs resulted in rapid clearance of HCV replicon RNAs.

Generally, siRNA design targeting the HCV genome is mainly based on only one design tool. For example, Watanabe *et al*. used the Ambion siRNA Construction Kit to design siRNAs targeting the HCV 5′ and 3′NTRs and the NS5B coding region, with good antiviral efficacies. Our *in silico*-designed siRNAs were even more efficacious. This result suggests that the use of state-of-the-art RNAi design tools can further improve potency and specificity [83]. Parabhu *et al*. used web-based Oligoengine software to design siRNAs targeting the 5′NTR of HCV, and revealed efficacy in Huh-7 cells transiently transfected with full-length genotype 1a and 1b clones [84,85,86]. Various siRNAs targeting the HCV 5′NTR were evaluated in a recently published study and the results indicate that HCV321 siRNA is one the most efficient siRNAs inhibiting HCV RNA replication [87]. Consistent with our results, the authors found that RNAi most efficiently inhibits viral replication when the HCV genome between base pairs 350 and 360 is targeted. They also found that repeated treatment combining two siRNAs targeting the IRES element can prevent the emergence of resistant HCV replicon cells. However, repeated treatment using a single siRNA leads to viral resistance. The authors also provided proof of concept by including an *in vivo* efficacy study using siRNAs packaged in nanosomes injected into the area of a subcutaneously formed hepatocellular tumor in a xenograft mouse model. A major hurdle still remains to efficient delivery of siRNAs specifically to target cells *in vivo* without adverse effects. Intensive research work has been performed to overcome these challenges. For example, modified gold nano particles with PEI have been applied to deliver successfully anti liver cancer siRNA[88]. Also, viral vectors with liver tropism, lipid nanoparticles containing ionizable amino lipids coated with polyethylene glycol to increase stability and prevent aggregation, or cholesterol-tagged and cell penetrating peptides linked to siRNAs are being used as vehicles for specific delivery to the liver [89]. The readiness of RNAi technology for use in patients was demonstrated in 2008 with the first FDA approval for the prevention and treatment of delayed graft function in kidney transplant patients. More recently, micro RNA 122 (miR-122), which is crucial for the RNA replication of HCV, became a drug target [90]. A recent phase 2 study revealed that when administered by subcutaneous injection, a siRNA targeting miR-122 (miravirsen) efficiently reduces viral load in the serum (NCT01200420).

In conclusion, we devised an improved *in silico* experimental triage process to design efficacious and specific siRNAs that targeted highly conserved regions of HCV genomes. Antiviral potencies were systematically assessed in HCV genotype 1 and 2 replicon cell lines and with a patient-derived infectious HCV genotype 4 isolate. Our results confirmed the predicted outstanding antiviral potency of the selected siRNA and a high barrier to resistance. Our *in silico*-designed siRNA might be a valuable antiviral agent and could be considered for further development as a chronic hepatitis C therapy.

## Supporting Information

S1 Table Important features and parameters of the 90% filtered set of siRNAs. (DOC)
