# Supplementary material for: In Silico Design and Experimental Validation of siRNAs Targeting Conserved Regions of Multiple Hepatitis C Virus Genotypes
Source: PLoS One. 2016 Jul 21;11(7):e0159211. doi: 10.1371/journal.pone.0159211 (PMC4956106; doi:10.1371/journal.pone.0159211)
Supplement: S1 Table — (DOC) [file pone.0159211.s001.doc]

**Supplementary Table 1:** Important features and parameters of the 90% filtered set of siRNAs.

|  |  | **Thermodynamic properties** | | | | **Scoring** | | | |
| --- | --- | --- | --- | --- | --- | --- | --- | --- | --- |
| **Position** | **Antisense** | **Sec. ΔG** | **5’ end** | **3’ end** | **Whole ΔG** | **s-Biopredsi** | **i- Score** | **DSIR** | **Target mRNA change %** |
| **258** | UUUCGCGACCCAACACUACuc | -1.9 | -2.2 | -0.9 | -38.7 | 0.767 | 64.8 | 84.6 | 1.99 |
| **353** | UUUUCUUUGAGGUUUAGGAuu | 2.4 | -2.4 | -0.9 | -32 | 0.845 | 69.4 | 88 | 3.89 |

Most specific and potent siRNAs with no near-perfect or miRNA-like off-target matches; while most potent implies best thermodynamic and target accessibility features such as differential end instability, and lack of palindromes, etc.
